# Supplementary figures and images for: Variation of mutational burden in healthy human tissues suggests non-random strand segregation and allows measuring somatic mutation rates
Source: PLoS Comput Biol. 2018 Jun 7;14(6):e1006233. doi: 10.1371/journal.pcbi.1006233 (PMC6007938; doi:10.1371/journal.pcbi.1006233)

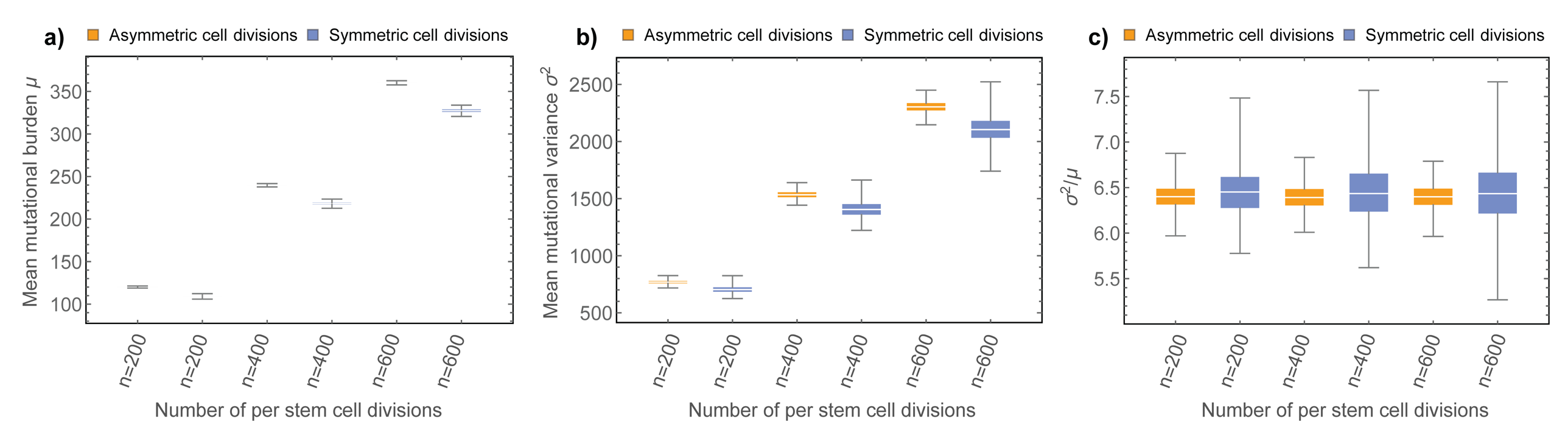

Supplement: S1 Fig — a) Mean mutational burden μ, b) mutational variance σ2, and c) the ratio of mutational variance and mutational burden σ2/μ for purely asymmetrically or a mix of symmetrically and asymmetrically dividing stem cells. Here we compare stochastic simulations for N = 5000 purely asymmetrically dividing stem cells with a strand segregation probability of p = 0.9 and stem cells with perfect strand segregation p = 1 but a fraction of 10% of stem cell divisions being symmetric differentiations followed by symmetric self-renewals. Both scenarios lead to a linear increase of mean and variance of mutational burden with minimal rate differences. However, as predicted, the ratio of variance and mean become time independent and are the same on average for both processes. However, the variance of the distribution of the ratio of the variance and mean increases with time for symmetric stem cell divisions but is approximately constant for asymmetric stem cell divisions. This effect might provide a future method to distinguish and quantitate the amount of symmetric self-renewal in human stem cell populations. (TIFF) [file pcbi.1006233.s001.tiff]
